# From microbial gene essentiality to novel antimicrobial drug targets

**DOI:** 10.1186/1471-2164-15-958

**Published:** 2014-11-05

**Authors:** Fredrick M Mobegi, Sacha AFT van Hijum, Peter Burghout, Hester J Bootsma, Stefan PW de Vries, Christa E van der Gaast-de Jongh, Elles Simonetti, Jeroen D Langereis, Peter WM Hermans, Marien I de Jonge, Aldert Zomer

**Affiliations:** Radboud Institute for Molecular Life Sciences, Laboratory of Paediatric Infectious Diseases, Radboud University Medical Centre, Nijmegen, 6500 HB The Netherlands; Radboud Institute for Molecular Life Sciences, Centre for Molecular and Biomolecular Informatics, Radboud University Medical Centre, Nijmegen, 6500 HB The Netherlands; NIZO food research, Ede, 6710 BA The Netherlands; Department of Veterinary Medicine, University of Cambridge, Cambridge, CB3 0ES UK; Crucell – Johnson and Johnson, Leiden, The Netherlands

## Abstract

**Background:**

Bacterial respiratory tract infections, mainly caused by *Streptococcus* pneumoniae, *Haemophilus influenzae* and *Moraxella catarrhalis* are among the leading causes of global mortality and morbidity. Increased resistance of these pathogens to existing antibiotics necessitates the search for novel targets to develop potent antimicrobials.

**Result:**

Here, we report a proof of concept study for the reliable identification of potential drug targets in these human respiratory pathogens by combining high-density transposon mutagenesis, high-throughput sequencing, and integrative genomics. Approximately 20% of all genes in these three species were essential for growth and viability, including 128 essential and conserved genes, part of 47 metabolic pathways. By comparing these essential genes to the human genome, and a database of genes from commensal human gut microbiota, we identified and excluded potential drug targets in respiratory tract pathogens that will have off-target effects in the host, or disrupt the natural host microbiota. We propose 249 potential drug targets, 67 of which are targets for 75 FDA-approved antimicrobials and 35 other researched small molecule inhibitors. Two out of four selected novel targets were experimentally validated, proofing the concept.

**Conclusion:**

Here we have pioneered an attempt in systematically combining the power of high-density transposon mutagenesis, high-throughput sequencing, and integrative genomics to discover potential drug targets at genome-scale. By circumventing the time-consuming and expensive laboratory screens traditionally used to select potential drug targets, our approach provides an attractive alternative that could accelerate the much needed discovery of novel antimicrobials.

**Electronic supplementary material:**

The online version of this article (doi:10.1186/1471-2164-15-958) contains supplementary material, which is available to authorized users.

## Background

The World Health Organization (WHO; http://www.who.int) ranks respiratory tract infections (RTI) among the ten leading causes of global mortality. RTI are associated with several bacterial species, of which *Streptococcus pneumoniae*, *Haemophilus influenzae*, and *Moraxella catarrhalis* are the most prevalent community-acquired respiratory bacterial pathogens [1]. In healthy individuals, these species colonize mucosal surfaces of the upper airways in a commensal state. Their relevance as pathogens arises when they infiltrate and colonize the otherwise sterile spaces in the middle ear, lung or bloodstream, progressing to disease [2]. With the mounting inexorable resistance of these pathogens against several commonly used antimicrobials [1], discovery of new protein targets against which new antibiotics could be developed will highly benefit global healthcare management of RTI.

Elucidation of genes essential for bacterial growth and viability is a prerequisite for identifying potential drug targets [3]. Essential genes are highly conserved and are thus considered as favourable drug targets for broad-spectrum inhibition [4]. On the other hand, some metabolic pathways constitute crucial transport and catalytic proteins which could also form attractive drug targets. Furthermore, most pathogens have drastically reduced their biosynthetic capabilities, and instead rely on their hosts to provide vital nutrients like amino acids, vitamins, and nucleobases [5]. Transport systems for these nutrients are generally conserved and indispensable for survival of the pathogen in its host [6], making them promising drug targets. In order to qualify as drug targets, microbial genes should meet several requirements. First, they should be nonhomologous to human genes to avoid drug cytotoxicity [3]. Additionally, targets should either be completely absent, or catalytically distinctive from genes found in host gut commensal microbiota, whose perturbation is likely to be detrimental to human nutrition, health, and physiology [7]. It has been shown that antibiotic killing of commensal microbiota facilitates proliferation, and often dominance, of antibiotic-resistant pathogens on mucosal surfaces [8]. Lastly, candidate drug targets must be accessible by inhibitors. Essential surface/membrane and secreted proteins are particularly promising, having been successfully targeted by protein drugs, and representing majority of all known drug targets [9, 10].

Previous microbial gene essentiality predictions employed techniques generally limited in specificity and/or throughput [11, 12]. These shortcomings are alleviated by high-throughput transposon insertion sequencing strategies, such as Tn-seq, TraDIS, INseq, or variants thereof, which have been applied in recent studies to comprehensively essay gene essentiality and genetic interactions in various bacteria [13, 14]. Here, we applied Tn-seq to reliably identify essential genes in *S. pneumoniae*, *H. influenzae* and *M. catarrhalis*. Products of these genes were compared against the human proteome, and the catalogue of genes from human gut commensal microbes, to identify and eliminate targets likely to have off-target effects in the host or on the host’s gut microbiota. Two out of four of the finally identified novel drug targets have been successfully validated using existing inhibitors. This study pioneers an integrative approach for rapid and cost-effective identification of novel drug targets. Our findings do not only improve the overall understanding of respiratory pathogens, but also serve as a proof of concept for the robust yet underexploited approaches, combining *in silico* and wet laboratory analyses in identifying antimicrobial drug targets, as recently reviewed [15]. This approach has allowed us to identify promising drug target leads, which after experimental validation could be potentially advanced to the discovery of novel antimicrobials for the treatment of RTI.

## Methods

### Bacterial genomes and gene reannotation

Whole genome sequences for *S. pneumoniae* TIGR4 uid57857, *S. pneumoniae* R6 uid57859, *H. influenzae* Rd KW20 uid57771, *H. influenzae* 86 028NP uid58093 and *M. catarrhalis* BBH18 uid48809 were obtained from the National Centre for Biotechnology Information (NCBI) Genbank File Transfer Protocol (FTP) website (ftp://ftp.ncbi.nih.gov/genbank/). All open reading frame (ORF) annotations were updated using Rapid Annotation using Subsystem Technology (RAST) [16]. In this analysis, all locus coordinates in original Genbank genomes release were retained without adjustments for frame-shifts.

### Orthology and gene essentiality predictions

We clustered the reannotated protein sequences into putative orthologous groups using the OrthoMCL standalone software Version 2.0.2 [17]. Most studies have consistently deciphered essential genes under ideal conditions, that is, in the richness of all necessary nutrients and without environmental stress. For the purpose of this study, we define the “essentiality” of a gene as its indispensability under rich media conditions, unless stated otherwise. The caveat with this approach is that essential genes required for metabolism within the host may be missed.

Transposon mutant libraries used were either created in-house for this study, or obtained from literature and reanalysed. The *M. catarrhalis* BBH18 *marinerT7* transposon mutant libraries consisting of 28,000 and 7,000 independent transformants were previously described [18, 19], and the 12,500 transformants library was generated using the previously described protocol [18]. The 40,000 transformants *S. pneumoniae* R6 and the 11,000 transformants *H. influenzae* 86 028NP library were previously described [20, 21]. Libraries for the 15,000 transformants *S. pneumoniae* R6 and *H. influenzae* Rd KW20 were also respectively constructed as previously described [20, 21]. The Tn-seq technology was used to profile the relative abundance of each mutant in all libraries after growth as described previously [22], except for *S. pneumoniae* TIGR4. Tn-seq data for *S. pneumoniae* TIGR4 were obtained from literature [23]. We then performed essentiality predictions for individual genes using the in-house developed web-tool, ESSENTIALS [24], which enabled us calculate a statistical essentiality metric for each ORF, and precisely delineate the optimal boundary between essential and nonessential ORFs in each of the 5 strains. Analysis data can be found at http://bamics2.cmbi.ru.nl/websoftware/essentials/links.html.

### Overrepresented metabolic pathways and subsystems

Pathways and subsystems for the strains under study were obtained from the Kyoto Encyclopedia of Genes and Genomes orthology, and the SEED databases respectively [25, 26]. Using a Fisher’s exact test, we performed functional categories enrichment for the pathways and subsystems, while incorporating the statistical essentiality value (the fold-change value predicted by ESSENTIALS) for each ORF. We corrected for multiple testing using Bonferroni correction and obtained *q-*values for corresponding *p-*values [27].

### Proteins subcellular localization (SCL)

The subcellular localizations (SCL) of all proteins in this study were determined using publicly available SCL prediction tools. First, we analysed all Gram-positive and Gram-negative strains using pSORTdb version 2.0 [10] and CELLO version 2.5 [28]. Further complementation SCL predictions were performed using LocateP and GnegPloc for Gram-positive and Gram-negative strains respectively [29, 30]. Additionally, the presence of integral Gram-negative outer membrane proteins (OMP) was determined using β-barrel outer membrane protein predictor (BOMP) [31]. Proteins that showed different SCL predictions in the different predictors used were denoted “Unknown”, together with those predicted to be of unknown SCL by majority of the predictors used.

### Selecting potential drug targets

To identify and eliminate essential genes with close undesirable orthologs, we performed separate unidirectional protein-protein BLAST (BlastP) searches, using an *E-*value cut-off of 1e-10, and minimum 70% sequence identity over 75% sequence coverage; against the human genome, and the metagenomics catalogue of non-redundant human gut microbiome genes by Qin *et al.* [7].

### Determination of antimicrobial activity

Selection of potential drug targets for *in vivo* validation was mainly based on their novelty, that is, they have not been described as targets to existing antimicrobials. Commercial availability of inhibitory compounds without resorting to customized chemical synthesis was also key; all inhibitory compounds used were supplied by Sigma Aldrich. 1-Methyluric acid, 5, 5′-Dithio-bis-(2-nitrobenzoic Acid), and 5′-deoxyadenosine were dissolved in water at 5 mg/ml. When necessary, the pH was neutralized (to pH7) using 10 M NaOH solution or 1 M HCl. Antimicrobial activity of the compounds was tested by Kirby-Bauer/disk diffusion assay [32], by applying 10 μg of the inhibitory compounds to 6 mm filter paper discs at concentration ranging from 10000 to 0.05 μg/ml in 10-fold stepwise dilutions. As for (R)-6-fluoromevalonate diphosphate 2 μl of (R)-6-fluoromevalonate diphosphate was diluted in 1 ml of Milli-Q (MQ). 10 μl and 100 μl of the dilution was used in separate disk diffusion assays. Columbia III agar with 5% sheep blood medium was used for *S. pneumoniae*. Brain heart infusion (BHI) agar medium, and a combination medium of BHI, hemin, and NAD were used for *M. catarrhalis* and *H. influenzae* respectively. MIC calculations were performed as described by Wiegand and colleagues [33]. Experiments were performed in quadruplicate, and outliers were removed using the Grubbs test [34].

### Toxicity assays on epithelial cell lines

Cellular toxicity of (R)-6-fluoromevalonate diphosphate was tested using the CellTox Green Cytotoxicity Assay (Promega, WI) on Detroit 562 (ATCC CCL-138) and A549 (ATCC CCL-185) cell lines according to the manufacturer’s instructions. The two cell lines were exposed to (R)-6-fluoromevalonate diphosphate at its effective MIC concentration of 26.6 μg/ml for 24 hours at 37°C with 5% CO_2_. Fluorescence was measured on a Perkin Elmer 1420 Victor 3 V multi-label plate reader.

## Results and discussion

### Genome reannotation and gene clustering

**Figure 1:**
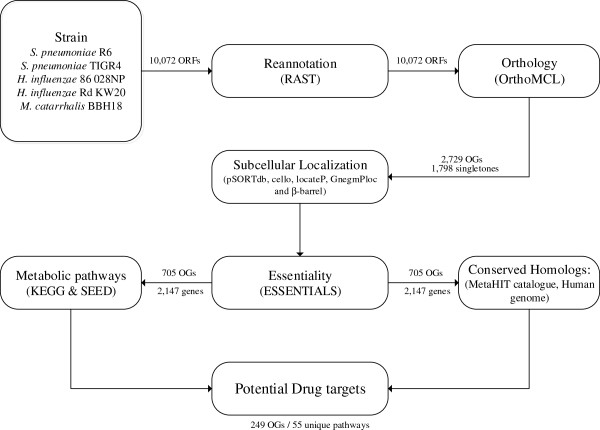
**Schematic overview of the drug target selection criteria.** Genome annotations information for *S. pneumoniae* R6, *S. pneumoniae* TIGR4, *H. influenzae* 86 028NP, *H. influenzae* Rd KW20, and *M. catarrhalis* BBH18 were updated using RAST. The proteins with updated annotations were then clustered into putative orthologous groups using OrthoMCL, and their subcellular localizations predicted in various publicly available tools. ESSENTIALS was used to analyse various transposon mutant libraries and predict the essentiality metric for each ORF. Comparing the ensuing essential genes with the catalogue of human gut microbial genes, as well as with the human genome helped to eliminate genes with conserved orthologs, and subsequently prioritize potential drug targets.

We sought to determine potential drug targets in *S. pneumoniae*, *H. influenzae*, and *M. catarrhalis* following the selection criteria outlined in Figure 1. For these species, five strains with the required Tn-seq data were available; *S. pneumoniae* strains R6 and TIGR4, *H. influenzae* strains Rd KW20 and 86 028NP, and *M. catarrhalis* strain BBH18. Altogether, genomes of these strains in their initial annotations constituted of 10,072 open reading frames (ORFs). These annotations were updated using RAST to ensure consistency and comparability among strains in subsequent analyses. This analysis resulted in putative annotations for about 50% of all ORFs originally annotated with a hypothetical function (Table 1; Additional file 1). Next, we clustered the updated protein sequences using OrthoMCL, producing 1,798 orthologous groups/clusters (OGs) with, and 2,729 without singletons respectively (Additional file 1). This clustering of orthologous proteins allowed for the determination of species and/or strain specific proteins, as well as determining the metabolic potential of the strains. For example, the “Gram-negative specific” periplasmic chaperones (SurA) were clustered in OG_756 (cluster 756), while the “Streptococci-specific” transcriptional regulators (LytR) were clustered in OG_2554. On the other hand, 300 OGs, including OG_184, OG_186, OG_216, and OG_224, among others, contained genes conserved in all the five strains. All protein in individual OGs constituted of similar or identical functional annotations. This consistency in grouping and annotation was observed across all OGs, suggesting a reliable clustering. Confirmatory clusters and respective annotations derived from the clusters of orthologous genes (COGs) database were consistent with our OrthoMCL clusters. Additionally, using the OG’s, we were able to curate annotations for the HI1586 locus in *Haemophilus influenzae* Rd KW20, which was possibly misannotated in the initial release, as an isoleucyl-tRNA synthetase instead of a NaP^+^P/HP^+^P antiporter.

**Table 1:** **Strain genome annotation updates and essentiality predictions**

			Annotations update		Essentiality predictions				
Strain	Genbank accession	Total number of ORFs	ORFs with hypothetical function in genome	ORFs with hypothetical function after RAST	Number of insertion sites ^***a***^	Log _2_fold change cut-off ^***b***^	Mutant library size (CFU)	Number of sequenced reads ^***c***^	Total essential genes
*S. pneumoniae* R6	NC003098	2,116	735	362	133,135	-6.45	40,000	8,906,301	325
								4,400,836**	
							15,000*	5,641,892*	
								6,335,218*	
*S. pneumoniae* TIGR4	NC003028	2,302	738	458	141,459	-4.43	6 × 20,000	876,181	414
								855,535	
								825,675	
								1,294,187	
								1,241,843	
								1,291,425	
*H. Influenzae* 86 028NP	NC007146	1,900	456	233	138,229	-4.64	11,000	5,751,765	532
								4,880,492	
								9,925,569	
								9,517,400	
*H. influenzae* Rd KW20	NC000907	1,790	429	118	131,955	-4.59	20,000*	3,857,040*	431
								3,229,286*	
								8,152,867*	
								7,724,536*	
*M. catarrhalis* BBH18	NC014147	1,964	586	573	116,242	-4.70	28,000	3,522,998**	445
							12,500*	4,618,913*	
							7,000	4,697,209	

Transposon mutant libraries and Tn-seq data prepared for this study (*), or Tn-seq data sequenced in this study from mutant libraries obtained from literature (**); otherwise, all data was obtained from literature and reanalysed in this study.

^*a*^Total number of possible unique transposon insertion sites in the genome; ^*b*^the computed fold change cut-off that separates essential and nonessential genes in each strain; ^*c*^number of sequence reads generated by the Illumina HiSeq sequencer.

### Essential and conserved protein-coding genes

Loss of mutant readouts from a transposon library after *in vitro* transposition and genetic transformation of the wild-type isolate is a strong indicator of gene essentiality [35]. Although some essential genes tolerate disruptive insertions in the 3′ regions, generally, insertions in essential genes lead to lethal phenotypes [36]. For our analysis, mutant libraries and/or Tn-seq data were constructed in in-house experiments or obtained from literature (Table 1). We separately analysed the Tn-seq datasets using ESSENTIALS [24]. This analysis resulted in the identification of 532 essential genes in *H. influenzae* 86-028NP, representing 28% of the genome, a higher number as compared to the other Gram-negative strains; *H. influenzae* Rd KW20 and *M. catarrhalis* BBH18, in which we identified 431 and 445 essential genes respectively. In *S. pneumonia*, we identified 325 and 414 essential genes for the R6 and TIGR4 strains respectively (Table 1; Additional file 1). These values showed that on average, about 20% of all genes in the five strains are essential. This is consistent with earlier studies which have reported 15-25% of all genes in a genome being essential [23, 36, 37].

Differences in the number of essential genes could be explained by various factors that hamper precision in transposon mutagenesis experiments, including short gene lengths and unsaturated transposon libraries; “saturation” being the presence of at least one insertion in every gene. In practice, short genes are less susceptible to disruptive transposon insertions, hence, more likely to be misclassified as essential. In unsaturated transposon mutant libraries, dispensable genes are also more likely to be devoid of transposon insertions, and therefore misclassified as being essential genes. The low-density transposon mutant library (approximately 11,000 colony forming units; CFU) used for *H. influenzae* 86-028NP, and a substantial number of short genes in its genome could, therefore, explain the apparently overestimated (532) essential genes. Relatively saturated libraries of approximately 20,000 CFU and 40,000 CFU were used for *H. influenzae* Rd KW20 and *M. catarrhalis* BBH18 respectively (Table 1). A rarefaction analysis on our data confirmed that the *S. pneumoniae*, *M. catarrhalis*, and *H. influenzae* Rd KW20 transposon libraries approached saturation (Additional file 2). Additionally, based on derivations of Poisson’s law, there is a 99.6% probability that genes with a size of 1 kb are hit in the 1.9 Mb *H. influenzae* 86-028NP genome and an 11,000 CFU mutant library. Similar statistics on the 1.79 Mb *H. influenzae* Rd KW20 genome with a 20,000 CFU mutant library shows a 99.99% probability. Therefore, *H. influenzae* 86-028NP could have suffered slightly more false positive predictions due to its less saturated mutant libraries.

**Figure 2:**
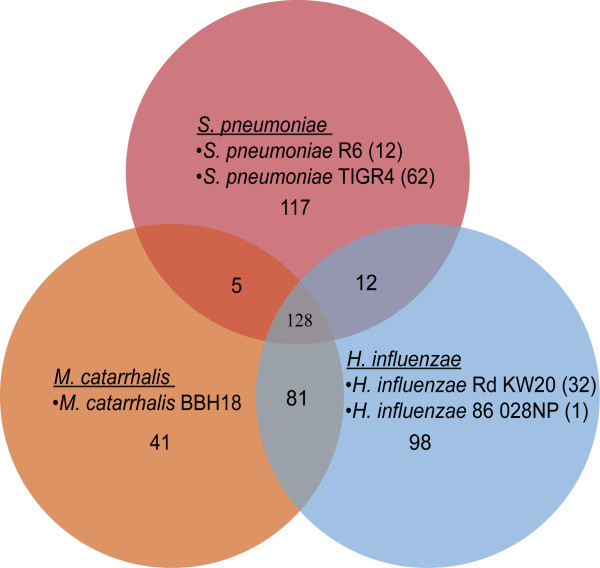
**A Venn diagram showing the overlap of essential orthologous groups among the respiratory pathogens.** Singletones are shown in brackets.

We selected 705 OGs containing at least one essential gene from any of the five strains for further analysis. These essential OGs mainly consist of proteins with annotated functions, participating in diverse core cellular processes, such as DNA replication, DNA transcription, protein translation, cell wall biosynthesis, signal transduction, and metabolism. Eighteen OGs, however, contained conserved proteins of uncharacterized function (Additional file 1). Functional characterization of these genes will aid in achieving the optimal set of targets that can be used to develop antimicrobials against RTI causing bacteria. The distribution and overlap of the essential genes within the three species is outlined in Figure 2. From the 705 OGs, we identified 128 OGs that constituted of genes conserved and essential in all five strains, representing targets particularly attractive for developing broad-spectrum antimicrobials to treat RTI, since they encode components of basal cellular functions in respiratory pathogens. Importantly, collective analysis of the five strains revealed species-specific and/or “Gram-category” specific essential genes, best suited for narrow-spectrum or specialized inhibition.

### Essential metabolic pathways and subsystems

Functional category enrichment analyses were performed for all KEGG metabolic pathways and the SEED subsystems [25, 26]. As of August 22, 2013, the KEGG database describes 448 fully characterized pathways, which are further subcategorized into 262,304 reference maps for various organisms. All KEGG characterized proteins in the 705 essential OGs could be assigned to 84 unique pathways. Among these, characterized proteins contained in the 128 OGs that are conserved and essential in all five strains could be assigned to 47 metabolic pathways (Additional files 1 and 3). As was the case for essential genes, the identified essential pathways specify among other functions, core bacterial bioprocesses like membrane transport, DNA replication and repair, signal transduction, metabolism, transcription and translation, ribosomal functions, and cellular processes including cell motility. The SEED, an alternative to KEGG, comprehensively groups genes at the level of a biological system and its subsystems. Currently, there are approximately 1,009 characterized SEED subsystems. Use of SEED subsystems on the essential OGs also revealed overrepresentation of critical system processes, including those involved in protein biosynthesis, virulence, disease and defence, as well as metabolism of cofactors, vitamins, prosthetic groups, pigments, fatty acids, lipids, and isoprenoids (Table 2; Additional file 4).

**Table 2:** **Distribution of essential features among respiratory pathogens**

	Quantity in the strain				
	mct	hin	hit	spn	spr
**Essential structural and non-coding RNAs**	**5**	**49**	**41**	**136**	**47**
tRNA	4	18	0	12	8
rRNA	1	31	41	44	30
sRNA	n/a	n/a	n/a	80	9
**Essential Protein-coding ORFs**	**445**	**431**	**532**	**414**	**325**
Protein of unknown functions	159	172	225	186	127
Metabolism	173	142	182	124	100
Genetic Information Processing	93	95	101	95	93
Environmental Information Processing	20	24	24	9	5
**Overrepresented/essential KEGG pathways**	**236**	**437**	**196**	**307**	**356**
Metabolism	136	221	95	171	213
Genetic Information Processing	74	177	74	128	129
Environmental Information Processing	26	38	26	8	14
Cellular Processes	0	1	1	0	0
**Overrepresented/essential SEED subsystems**	**449**	**513**	**602**	**450**	**355**
Protein metabolism	84	85	99	100	93
Cofactors, Vitamins, Prosthetic Groups, Pigments	75	61	80	29	25
Cell Wall and Capsule	47	60	78	47	30
Amino Acids and Derivatives	41	59	58	14	11
Respiration	41	16	34	8	7
Fatty Acids, Lipids, and Isoprenoids	29	36	40	26	21
RNA Metabolism	25	59	71	60	39
Carbohydrates	24	30	46	47	35
DNA Metabolism	19	37	35	45	41
Stress Response	18	17	9	10	8
Nucleosides and Nucleotides biosynthesis	17	13	11	25	9
Virulence, Disease and Defence	16	18	18	16	15
Regulation and Cell Signalling	8	4	8	6	5
Cell Division and Cell Cycle	5	18	15	17	16

The strains under study are abbreviated: mct; *Moraxella catarrhalis* BBH18, hin; *Haemophilus influenzae* Rd KW20, hit; *H. influenzae* 86 028NP, spn; *Streptococcus pneumoniae* TIGR4, and spr; *S. pneumoniae* R6. Untested categories are denoted by “n/a”.

### Protein subcellular localization

Out of the 705 OGs selected, the majority (526) consists of cytoplasmic proteins. Cellular localization of the other OGs were predicted to be: 96 in the inner membrane, 11 in the outer membrane, 12 in the periplasm, and 4 in the extracellular space. In addition, 21 OGs are non-categorically predicted to contain membrane proteins, whereas 35 are of unknown localizations. Of the 11 outer membrane OGs, 7 contained *β*-barrels (Additional file 1).

### Orthologs in human and human gut microflora

The human gut is home to microbiota whose proper composition and functioning collectively influence human nutrition, protection against pathogens and development of disease [7]. Perturbing this microbiota with antibiotics could cause adverse side effects. Furthermore, interference with human cell physiology by antibiotics as a consequence of non-specific targeting can cause severe cellular cytotoxicity [3], which may result in organ failure or even death. We used blastP analyses against the human genome (Genome Reference Consortium) and the human gut microbial gene catalogue [7], to identify targets that would likely have off-target effects. It is noteworthy that targets with as few as 10 matches in the non-redundant gut microbial gene catalogue were allowed in the final selection, as we hypothesised that these would have no effects on the gut microbiome preventing disruption of gut health. This decision was motivated by the observation from our analysis that well known targets for both clinically approved antimicrobials and experimental small molecule inhibitors collated in DrugBank (Additional file 1; column 9) maintained on average fewer than 10 blast hits against the human gut microbial gene catalogue (Additional file 1; column 20). On the other hand, the majority of the targets with numerous blast hits were aminoacyl-tRNA synthetases (aaRSs) and ribosomal protein, including rpsL, a well-known target that had 249 hits for pneumococci, 156 for *H. influenzae*, and 151 for *M. catarrhalis*. One shortcoming of using such filtering criteria is that novel targets that have more than 10 blast hits are not effectively retained in the final selection. Nevertheless, we identified 96 OGs with orthologs in human, and 127 OGs with orthologs in human gut microflora, that is, with >10 blast hits (Additional file 1). All 20 aminoacyl-tRNA synthetases (aaRSs), essential for protein synthesis, were particularly conserved in both human and human gut microflora. Studies have shown that aaRSs can be selectively targeted as most bacterial aaRSs recognize and aminoacylate only cognate tRNA [38]. However, possible side effects are expected from drugs targeting aaRSs. RNA molecules and ribosomal proteins were also highly conserved in gut microbiota and humans. Additionally, the relatively short lengths and the presence of highly repetitive DNA in RNA sequences also rendered their essentiality predictions unreliable. All these molecules were therefore not included in the final selection of drug targets. Moreover, blast comparison between finally selected targets and their human orthologs showed minimal sequence identities (<35%) over short sequence coverage.

### Drug targets selection and validation

We identified 249 potential drug targets in the five strains (Additional file 5), including key enzymes in pathways such as fatty acid biosynthesis [39–41], vitamin biosynthesis [42–45], and isoprenoid biosynthesis pathways [46–48], which have gained interest in drug discovery research, as well as 67 known targets inhibited by 75 FDA-approved antimicrobial drugs and 35 other researched small molecule inhibitors collated in the DrugBank database [49]. To validate our target prediction, we selected four novel targets with commercially available novel inhibitors of their predicted essential functions, that is, inhibitors not yet approved as clinical drugs and don’t require to be custom synthesized: We tested whether exposure to these compounds inhibited growth of the target organisms.

Vitamin biosynthetic pathways constitute an attractive and largely untapped source of potential drug targets [42, 45]. For instance, thiamine (vitamin B1) in its active form thiamine diphosphate, is indispensable for the activity of the carbohydrate and branched-chain amino acid metabolic enzymes [42]. Most bacteria synthesize thiamine *de novo,* whereas humans depend on dietary uptake, making the thiamine biosynthetic pathway an attractive selective drug target. Folic acid (vitamin B9) is another indispensable cofactor, whose biosynthetic pathway was a target for sulfamidochrysoidine (prontosil); later replaced by an improved sulphonamide drug sulfanilamide, the first ever antibiotic used in humans [50]. The pathway is also targeted by trimethoprim [45], another clinically acknowledged chemotherapeutic agent that acts on dihydrofolate reductase. Niacin (vitamin B3; alternatively known as nicotinamide or nicotinic acid) is also essential to all living cells and is biosynthetically converted to nicotinamide adenine dinucleotide (NAD^+^), a coenzyme involved in electron transport reactions in cell metabolism processes [51]. After it was described that niacin has therapeutic effects and it modulates various biological effects as well as NAD^+^ metabolism, there has been an increased interest in the role of NAD^+^ biosynthetic pathway in health and disease [52]. Prospects of targeting the pathway are also being explored. We used 5, 5′-Dithiobis, 2-nitrobenzoic acid (CAS: 69-78-3) to inhibit NAD^+^ kinase (EC: 2.7.1.23) - a key enzyme in the NADP biosynthesis which catalyses the phosphorylation of NAD^+^ into NADP^+^. Inhibition of growth was expected in both Gram-positive and Gram-negative strains. However, inhibition of growth was only observed in the disk diffusion assays for *S. pneumoniae*. MIC calculations were inconclusive as they ranged from 319 to 2500 μg/ml with a large variability between assays (Table 3).

**Table 3:** **Drug target in vivo validation summary**

Compound	Amount on disc (μg)	MIC μg/ml; Std. Dev. [Inhibition area on disk diffusion assay]		
		***S. pneumoniae***	***H. influenzae***	***M. catarrhalis***
5,5′-dithiobis(2-nitrobenzoate) (CAS 69-78-3)	1,000	2,500; 0 [4 mm*]	781; 313 [none]	319; 303 [none]
1-methyluric acid (CAS 708-79-2)	1,000	>312.5 [6 mm]	>312.5 [none]	>312.5 [none]
5′deoxyadenosine (CAS 4754-39-6)	1,000	78.1; 0 [6 mm]	205; 132 [5 mm*]	29.3; 11 [12 mm]
(R)-6-fluoromevalonate diphosphate (CAS 2822-77-7)	1,000	26.6; 11.5 [12 mm]	4,167; 1443 [none]	>5,000; 0 [none]
(R)-6-fluoromevalonate diphosphate (CAS 2822-77-7)	100	26.6; 11.5 [4 mm*]	4,167; 1443 [none]	>5,000; 0 [none]

Diameter of the clearance zone after normal incubation represents the inhibition area on disk. Concentrations showing delayed growth are denoted by an asterisk (*).

Std. Dev. = Standard deviation.

**Figure 3:**
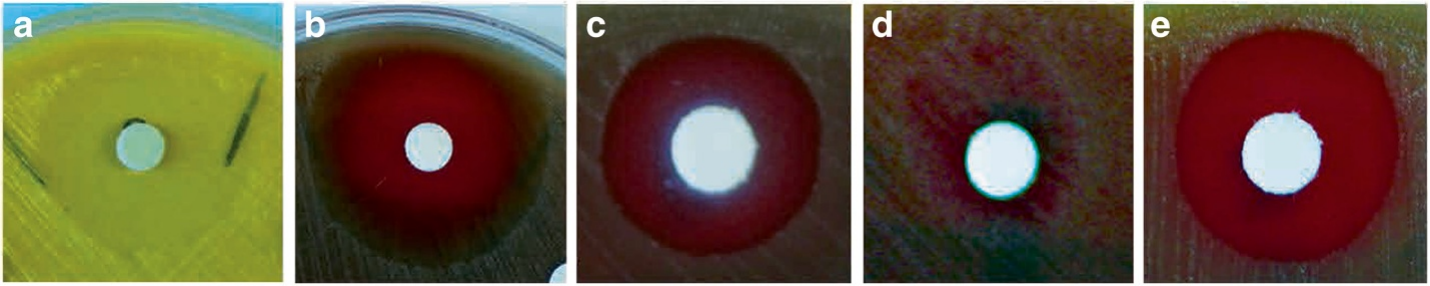
**Validation of growth inhibition using disk diffusion essays.** Cell culture plate cross-sectional images showing the area of growth inhibition for: **a**. *M. catarrhalis* in 5′deoxyadenosine, and *S. pneumoniae* in; **b**. (R)-6-fluoromevalonate diphosphate, 1-methyluric acid, **d**. 5, 5′-dithiobis (2-nitrobenzoate), and **e**. 5′deoxyadenosine respectively.

As an essential amino acid, methionine is not synthesized *de novo* in humans, who must rely on dietary intake. Enzymes involved in microbial methionine biosynthesis therefore offer highly specific and selective drug targets. We used 1-methyluric acid (CAS: 708-79-2) to target S-adenosylmethionine synthetase (EC: 2.5.1.6); a key enzyme in methionine biosynthesis, whose drug target potential has been explored in various pathogens [53, 54]. Contrary to expectations, no growth inhibition was observed in Gram-negative strains (Table 3; Figure 3): growth inhibition was only observed in *S. pneumoniae*. Since 1-methyluric acid formed a precipitate in concentrations above 312 μg /ml, no MIC values could be calculated. This lack of growth inhibition in Gram-negative strains may possibly be due to their double layered cell walls which are less penetrable [55], or the bacteria have expanded their resistance mechanisms to evade killing by antimicrobials [55, 56]. It is also possible that the two Gram-negative species have alternative mechanisms for methionine biosynthesis, further complicating screening for effective drugs.

The microbial fatty acid synthesis (FAS) pathway is an attractive target for drug discovery [41, 57]. This pathway is subdivided into type I and II, whereby human FAS proteins predominantly belong to type I FAS, and the bacterial ones are predominantly type II FAS. Proteins from the two FAS types generally possess distinctive molecular organization of the active site allowing for selective targeting [39, 40]. Although Gram-positive pathogens could compensate FASII inhibition by assimilating environmental fatty acids; particularly unsaturated fatty acids [58, 59], several clinical and household antimicrobials targeting key FAS enzymes, e.g. Platensimycin and Platencin have been successfully developed [41, 60]. In our analysis, we identified various genes conserved in all five strains, for example genes in OGs 085, 143, and 653, whose products play key roles in the FAS pathway. With 5′-Deoxyadenosine (CAS: 4754-39-6), we targeted lipoate synthase (LipA; EC: 2.8.1.8), a key enzyme in the lipoic acid metabolism [61], using product-level inhibition. Surprisingly, we observed growth inhibition in all three species (Figure 3; Table 3), despite the target cluster (OG_653) comprising of orthologs from only Gram-negative strains (Additional file 1). This observations are also reflected in the MIC, which ranged from 29.3 to 205.1 μg/ml (Table 3). A blastP comparison showed that the closest ortholog of the Gram-negative LipA in *S. pneumoniae* is the non-lipoic pathway enzyme fructose-6-phosphate aldolase I, sharing about 32% sequence identity. Moreover, a comparison between LipA and lipoate-protein ligase (LplA), the key lipoylation enzyme in *S. pneumoniae* [61], revealed that the two proteins are non-orthologous, as they share very low sequence identity (<25%). They however have conserved domain which may explain the observed growth inhibition.

Isoprenoids are natural products involved in many biochemical functions, such as supplying quinones for the electron transport chains, components of membranes, and subcellular targeting and regulation [47]. Humans employ the mevalonate pathway, whereas most microbes follow a non-mevalonate (1-deoxy-d-xylulose 5-phosphate/2-C-methyl-d-erythritol 4-phosphate) pathway. Functional roles of key enzymes in the isoprenoid biosynthesis pathway are well characterized, opening prospects for the discovery of novel drug targets [46, 48]. Fosmidomycin is a promising isoprenoid-based anti-malarial drug which is currently in clinical trials [48]. Using 6-fluoromevalonate (CAS: 2822-77-7) to target diphosphomevalonate decarboxylase (EC: 4.1.1.33), we observed selective growth inhibition only in *S. pneumoniae* as expected (Figure 3; Additional file 1; Table 3). Additionally, no effects on growth were observed in the Gram-negative strains, which was also as expected. We determined an average MIC of value 26.6 μg/ml for the *S. pneumoniae* growth inhibition (Table 3). At 26.6 μg/ml, no toxicity was observed in cell toxicity assays on epithelial cell lines (data not shown). Moreover, in patent WO 1995013058 A1, no cytotoxic effects of 6-fluoromevalonate were observed on T-lymphocytes. Previous literature also shown that 6-fluoromevalonate could potentially function the same as statins, as they inhibit the same pathway [62]. Diphosphomevalonate decarboxylase could therefore be a promising target for developing novel antibiotics against *S. pneumoniae* [63].

## Conclusion

We have combined Tn-seq with *in silico* approaches to obtain an insight into many essential and conserved molecular functions, which we predicted to be unique among respiratory pathogens. With this combinatorial approach, we have reliably identified 249 potential drug targets, 67 of which are acknowledged targets for 75 FDA-approved antimicrobial drugs and 35 other researched small molecule inhibitors [49]; we successfully validated two of the four tested targets. Here, we propose a number of novel potential drug targets that are a concrete lead for experimental validation. We anticipate that future research based on this study will eventually provide interesting targets that can be successfully moved to drug development. In conclusion, we have pioneered a powerful approach, which combines microbial gene essentiality data with robust computational techniques, to comprehensively screen for antimicrobial drug targets at genome-scale. This approach circumvents the complex and costly laboratory screens, thus, facilitating directed drugs discovery.

### Availability of supporting data

The data sets supporting the results of this article are included within the article and its additional files. Tn-Seq data sets are available in the European Nucleotide Archive repository, [http://www.ebi.ac.uk/ena/data/view/PRJEB7553].

## Electronic supplementary material

Additional file 1: **The essential clusters.** All clusters of orthologous genes that contained at least one essential gene. For genes in each cluster, information on the original Genbank and updated RAST annotations, known inhibitors and drugs, essentiality prediction metrics, subcellular localization, and potential availability of undesirable orthologs in genes from normal gut microbiota as well as the human host are collated. (XLSX 530 KB)

Additional file 2: **A line graph of the rarefaction analysis Reference** [13] **on the transposon mutant libraries used in study.** The probability that more essential genes are hit increases with the increase in mutant library saturation. (PDF 234 KB)

Additional file 3: **KEGG functional categories enrichment using fisher’s exact test.** Unrepresented categories are denoted by “n/a”. (XLSX 39 KB)

Additional file 4: **The SEED functional categories enrichment using fisher’s exact test.** Unrepresented categories are denoted “n/a”. (XLSX 70 KB)

Additional file 5: **A summary of all selected potential target.** This is a summarized version of Additional file 1. OG denotes the Orthologous cluster as determined using OrthoMCL (Additional file 1; reference 17). (XLSX 32 KB)
