# Supplementary material for: From microbial gene essentiality to novel antimicrobial drug targets
Source: BMC Genomics. 2014 Nov 5;15(1):958. doi: 10.1186/1471-2164-15-958 (PMC4233050; doi:10.1186/1471-2164-15-958)

Additional file 4| A line graph of the rarefaction analysis [Reference 13] on the transposon mutant libraries used in study. The probability that more essential genes are hit increases with the increase in mutant library saturation.

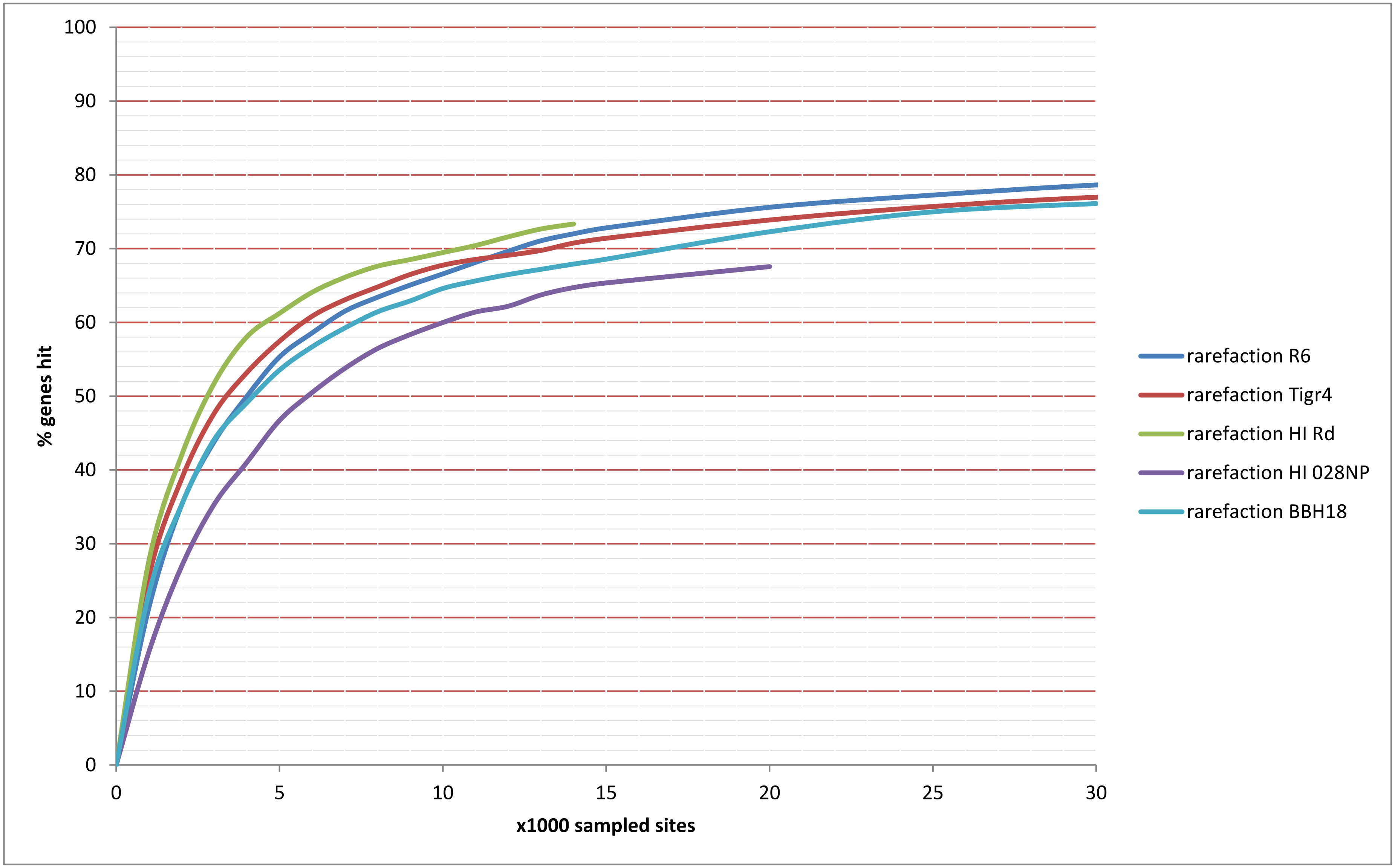

Supplement: Supplementary file 2 — Additional file 2: A line graph of the rarefaction analysis Reference [13] on the transposon mutant libraries used in study. The probability that more essential genes are hit increases with the increase in mutant library saturation. (PDF 234 KB) [file 12864_2014_6655_MOESM2_ESM.pdf]
